# Stroke unit and Neurocritical Care Unit for acute neurological diseases in the USL Toscana Centro: a pilot model of Santo Stefano Hospital in Prato

**DOI:** 10.3389/fstro.2023.1218682

**Published:** 2023-12-07

**Authors:** Raffaella Valenti, Alba Caruso, Anita E. Scotto Di Luzio, Donatella Accavone, Maria G. Cagliarelli, Guido Chiti, Enrico Grassi, Maria Briccoli Bati, Pasquale Palumbo

**Affiliations:** S.O.C. Neurologia, Neurofisiopatologia - Stroke and Neurocritical Care Units, Ospedale Santo Stefano, Azienda USL Toscana Centro, Prato, Italy

**Keywords:** acute neurological diseases, acute phases of neurological disease, stroke unit, neurocritical care, neurocritical care unit, expertise, emergency treatment, specialized dedicated setting

## Abstract

**Background:**

Acute neurological diseases are leading causes of disability and death. The need for specialist neurocritical care skills for managing neurological emergencies has increased. Promising opportunities exist to improve outcomes in acute phases of neurological diseases, such as, for example, the concept of a stroke unit for stroke patients. A similar concept was introduced for a neurocritical care unit, which is associated with improved clinical outcomes compared with more traditional management. However, neurocritical care is often not recognized as a separate specialty. Significant progress in neurology has enabled better approaches for the critically ill neurologic patient, in particular those with stroke and hemorrhage, but also with epileptic seizures and epileptic status, traumatic brain injury (TBI), subdural/epidural hematoma, acute inflammatory polyradiculoneuritis, encephalitis, myasthenia gravis, acute myelitis, etc.

**Assessment of policy/guidelines options and implications:**

Except for cerebrovascular diseases, for other acute neurological diseases, there is no standardized model care service recognized. A good patient outcome can be obtained by the choice of neurology setting for acute patients including nursing and medical staff with specific training in neurocritical care. As we believe neurocritical care practices should be implemented, we suggest a pilot model on the basis of our experience. In this report, we show a model of the 2A setting of the Santo Stefano Hospital (Prato, USL Toscana Centro), where, as well as stroke units for cerebrovascular events, we have neurocritical care unit and acute-neurology experts for all acute neurological diseases.

**Actionable recommendations: our pilot experience:**

The 2A setting of Stroke Unit/Neurocritical Care of the Santo Stefano Hospital includes 15 beds; 8 ± 2 beds are monitored by portable multi-parameter monitoring devices. Following acute treatment, diagnostic/etiologic work-up and automated monitoring of vital functions are performed in addition to adapted secondary prevention, early rehabilitation, and prevention of complications in all acute patients. We retrospectively assessed the diagnoses in the hospital discharge forms (HDF) of Stroke Unit/Neurocritical Care (2A) of 249 patients consecutively analyzed between 1 January 2022 and 30 June 2022. Out of the 249 patients affected by acute neurological diseases, 155 had cerebrovascular diseases (62.2%). In particular, 100 (64.5%) were diagnosed with ischemic stroke and 44 (28.4%) with hemorrhagic stroke. Thirty-two patients (12.8%) were hospitalized following a TBI. Sixteen patients (6.4%) had a new diagnosis of epilepsy and three (1.2%) of epileptic status. In our setting, the 3-month modified Rankin Scale (mRS) in the 36 stroke patients treated with recombinant-tissue plasminogen activator (r-TPA) was 0–2 (low disability) in 60% of cases. Additionally, 31% of 44 intracerebral haemorrhage (ICH) patients reported a moderate-severe degree of disability. Regarding TBI patients, the mRS ranged from 1 to 5, with significate difference between patients in Stroke Unit/Neurocritical Care (2A) in comparison with those in other departments (2–3 vs. 3–4, respectively). Globally, the setting discharge of the acute neurological patients were: rehabilitation (26%), intermediate care hospitals (44%), long-term care (5%), and home (25%). The 1-month mortality rate was 1.8%.

**Discussion:**

We provide a brief description of the cases treated over a 6-month period to draw attention to the possibility of the existence of a ward dedicated exclusively and specifically to all acute neurological diseases. The sample of patients is very varied and interesting. More than 60% of patients had cerebrovascular diseases. The paucity of outcome data makes this report limited, but the diagnostic-therapeutic strategies, the presence of staff trained in specific neurocritical care, and the use of inpatient hospital-based registries are, in our opinion, strengths. Our pilot model of the setting of Stroke Unit/Neurocritical Care (2A) in the Santo Stefano Hospital (Prato, USL Toscana Centro) should be further implemented, also to verify systematically the associations with measurable outcome improvements in patients affected by strokes and other neurological acute diseases.

## Introduction

### Background

Acute neurological diseases (AND) are among the major contributors to the problem of illness worldwide. In recent years, the global burden of AND, in particular cerebrovascular diseases and ischemic or hemorrhagic strokes, has increased (Prust et al., 2022). Stroke is the second leading cause of death worldwide (World Health Organization, 2018a). According to the Global Burden of Disease study, neurological diseases are the greatest driver of worldwide disability, with stroke accounting for nearly half of this disease burden (Prust et al., 2022). This is mainly due to the increase in the average age of the population, the greater presence of vascular risk factors, and the improvement in the treatment of diseases (World Health Organization, 2018a). AND [e.g., traumatic brain injury (TBI), epilepsy, and other pathologies in their acute phases], as cerebrovascular diseases, have important repercussions on social and economic costs (Prust et al., 2022). Acute neurological survivors experience a significantly greater affliction of long-term disability (Langhorne et al., 2018).

At the same time, the need for specialist neurocritical care skills aimed at managing neurological emergencies has increased, as well as the need for resources such as intensive monitoring beds, advanced neuroimaging, sophisticated neurointerventional skills, and drugs (Prust et al., 2022). Specialized neurocritical care is predominantly in high-income countries (Mateen, 2011; Prust, 2020) because of costly public health resources and investments (Prust et al., 2022). A recent paper reported more than 70 countries with less than five intensive care unit beds per 100,000 inhabitants (Ma and Vervoort, 2020); the number decreases in low-income countries (Phua et al., 2020). Often, medical doctors and health professionals specialized in the management of neurological diseases in the acute phase are missing (0.13–4.75 neurologists per 100,000 inhabitants) (Prust et al., 2022).

Therefore, it is necessary provide the best possible critical clinical care for patients with AND. For example, for patients with stroke, the concept of stroke unit care was conceived (Prust et al., 2022).

### The concepts of stroke units and neurocritical care units

The model of stroke unit care was born about 50 years ago, but its effectiveness has only been demonstrated in the last 20 years (Garraway et al., 1980; Ebrahim, 1990; Indredavik et al., 1991; Stroke Unit Trialists' Collaboration, 1997a,b; Langhorne, 2021). In fact, until recently, it was thought that the natural history of the stroke could not be changed (Weatherall et al., 1983; Langhorne, 2021).

The first description of a stroke unit setting was reported in the 1950s and 1960s (Langhorne, 2021). The concept was to focus stroke care around a multidisciplinary team of stroke specialists who operated out of a single specialized unit (the “*stroke unit*”), defined as incorporating a “multidisciplinary team of specialists in the care of stroke patients” (Langhorne et al., 1993; Langhorne, 2021). In 2020, the Cochrane Collaboration has demonstrated that stroke patients admitted to stroke units are more likely to survive and have less residual functional disability; the benefits remain independent of patient age, sex, initial stroke severity, or stroke type (Langhorne and Ramachandra, 2020). This evidence has resulted in recommendations reported in many national and regional guidelines (Stroke Foundation, 2002; Norrving et al., 2018; Langhorne and Ramachandra, 2020) that all stroke patients should be treated in a specific dedicated setting, such as stroke units. Some articles have highlighted the negative aspect of stroke units in terms of costs, thus questioning their effectiveness in terms of cost benefits (Mateen, 2011). For this and other reasons, the actual geographical distribution of stroke units, including in Italy, is patchy (Langhorne, 2021). More recent studies (Urimubenshi et al., 2017; Norrving et al., 2018) have shown an association between hospitalization in stroke units and better patients outcomes (Langhorne, 2021).

In the last 10–20 years, similar concepts were introduced for neurocritical care (Suarez, 2006). The birth of the concept of Neurocritical Care Unit was even more complicated than that of the Stroke Unit concept (Shrestha and Lamsal, 2020; Prust et al., 2022). Generally, neurocritical care was mainly for management of TBI, central nervous system infectious, and surgery patients, especially if in a serious clinical condition and with imminent risk to life. The establishment of neurocritical care units with acute phase neurointerventional treatments was associated with improved clinical outcomes compared with more traditional management (Varelas et al., 2004; Suarez, 2006; Kramer and Zygun, 2014; Shrestha and Lamsal, 2020). The possible reasons for better outcomes included: dedicated physicians and nurses, protocolized management, and stricter adherence to neurocritical care protocols (Shrestha and Lamsal, 2020). Nevertheless, neurocritical care is often not recognized as a separate specialty and is restricted to a few large academic institutions with a shortage of neurocritical care beds and common neurocritical care modalities (Shrestha and Lamsal, 2020).

### Acute neurological diseases

Intracerebral hemorrhage (ICH), the second most common and more deadly cause of stroke (15–30% of strokes), with a one-year mortality around 50% and 5-year survival <30% (Sporns et al., 2021; Prust et al., 2022), has resulted in the urgent need to develop new strategies to treat and try to stop ICH in its hyperacute phases (World Health Organization, 2018b; Sporns et al., 2021). Although there is no specialized medication for ICH, there are some innovative approaches for the acute management and recovery. The main therapeutic strategies, according to guidelines, are strict blood pressure control (in ICH due to small vessel disease), and endovascular treatment with embolization (in ICH due to arteriovenous malformations or aneurysm). The implementation of multimodal imaging allows timely etiological diagnosis and the assessment of early hematoma expansion, also with a view to therapeutic intervention and evacuation surgery (Sporns et al., 2021). ICH patients are generally hospitalized in intensive care units (not always with neurological care settings) or in neurosurgery.

In modern times, there has been a sharp increase in TBI due to the increase of motor vehicles (Bannick et al., 2019). Road accidents cause ~1.3 million deaths each year and are among the leading causes of death worldwide, particularly in young people (Bannick et al., 2019). In addition, in patients over the age of 70, the trauma related to falls is very frequent with consequent post-traumatic (subdural and/or epidural) hematoma (Dewan et al., 2018). As the global population ages, the incidence of fall-related TBI continues to increase, especially with substantial gains in life expectancy in recent decades (Dewan et al., 2018; Bannick et al., 2019). Some studies have demonstrated the advantages of neurocritical care units and effect of a specialized neurocritical care team in monitoring and treating TBI patients (Varelas et al., 2004; Suarez, 2006; Kramer and Zygun, 2014; Shrestha and Lamsal, 2020).

Other AND to be taken into account for the severe impact on the neurocritical care include: polyradiculoneuritis (infectious, inflammatory, or autoimmune), encephalitis, epileptic seizures, acute phases of myasthenia gravis, and myelitis (Prust et al., 2022).

The global burden of epilepsy and epileptic status is mainly secondary to dystocic delivery at birth, central nervous system infections, and structural causes, such as traumatic and non-traumatic brain injuries (vascular, neoplastic, etc.). In case of epileptic seizures (especially of first epileptic seizures), access to electroencephalogram (EEG) and neuroimaging for early diagnosis is very important. Rapid access to neurophysiological investigation, as continuous EEG, is required to detect, monitor, and treat critical non-convulsive epileptic status (Prust et al., 2022). Treatment of convulsive and non-convulsive epileptic status includes both management and prompt cessation of seizure activity and the earliest possible detection of an underlying etiology for specific acute treatment (Trinka and Leitinger, 2022). In the real life continuous EEG monitoring is often missing, also because there are no dedicated beds.

Furthermore, beyond acute phase management, neurological expertise, coupled with neurophysiological expertise, also allows early patient prognostication with stratification of patients both for the purposes of more or less aggressive treatment and for the definition of an appropriate rehabilitation pathway (e.g., acquired brain injured patients, epilepsy, etc.).

In addition to AND, there are systemic complications of acute phases of neurological patients: fever, glycemic and blood pressure decompensations, infectious and sepsis, pneumonia, dysphagia, systemic thromboembolism, respiratory failure, and shock (Prust et al., 2022). For a better outcome, patients should undergo regular monitoring of vital signs and correction of hypertension and diabetes, as well as the treatment of fever, correction of electrolyte imbalances and hypoxemia, early mobilization, and prevention of thromboembolism and dysphagia while avoiding complications (Prust et al., 2022).

## Assessment of policy/guidelines options and implications

While patient care is becoming more complex, the challenge is to match the health system with the necessities of the patients (Langhorne et al., 2020; Langhorne, 2021), providing the best pathways and the best possible assistance according to the needs of the patient.

In recent years, important progress has been made in the management and treatment of AND. For patients affected by ischemic stroke, for example, a number of interventions have been developed particularly for early reperfusion in cerebral vessels (Langhorne et al., 2020), including the application of reperfusion therapy with a thrombolytic drug and/or mechanical thrombectomy. Many services are being re-shaped with the aim of providing emergency thrombolysis and/or thrombectomy for as many patients as possible and in the shortest possible time (Ringelstein et al., 2013). This has resulted in the development of service models (such as comprehensive stroke centers and primary stroke centers, called hub- and spoke- centers) where the primary focus is hyperacute interventions with subsequent stroke unit model care (Ringelstein et al., 2013; Man et al., 2018).

Unfortunately, for other AND, there is no standardized model care service recognized. The early stabilization of patients with AND in a dedicated setting is crucial. As well as stroke patients, patients affected by other AND require skilled and continuous nursing care in a specific and dedicated setting, multidisciplinary assistance for the prevention of complications, early treatment of infections and seizures, and early rehabilitation with discharge planning and possible continuing rehabilitation after returning home (Langhorne, 2021). Moreover, there are many other new therapies and approaches that have been introduced for the early phases of AND (Suarez, 2006).

In the inpatient setting, limited access to basic critical care resources and neurologic expertise means that most neurologic emergencies are managed on low-acuity wards by staff who have received limited focused training in care for acute neurological disease (Dart et al., 2017; Norrving et al., 2018; Banerdt et al., 2021). The care of patients with AND can vary considerably between countries and between healthcare settings due to local epidemiology, affordability of care, health system financing, and resource availability (Prust et al., 2022). Thus, the good practices must respond to the complexity of local epidemiology and the availability of local resources.

The choice of neurology setting for acute patients with nursing and medical staff with specific training in neurocritical care and with acute neurologic expertise is fundamental for patients' outcomes (Varelas et al., 2004; Suarez, 2006; Kramer and Zygun, 2014; Shrestha and Lamsal, 2020). There is significant evidence in the literature indicating the better quality assistance that stroke units provide in the treatment of stroke (Langhorne and Ramachandra, 2020; Prust et al., 2022). There are also some few studies on neurocritical care units, focused on TBI, infections, and post-traumatic hemorrhage; patients had better outcomes when managed in neurocritical care units rather than conventional neurology or neurosurgical departments (Varelas et al., 2004; Suarez, 2006; Kramer and Zygun, 2014; Shrestha and Lamsal, 2020). The specific neurology setting is missing.

Standardized service models and standardized neurocritical care practices should be implemented also for all AND, as well as stroke units for cerebrovascular events.

In this paper, we suggest a pilot model of neurocritical care units specific for all AND.

## Actionable recommendations

In recent years, the Azienda USL Toscana Centro in Tuscany (Italy) has witnessed the birth of the care model defined by “*intensity of care*”, in which three levels of care are provided [critical care (Level 1), high care (Level 2), and low care (Level 3)]. Level 2 includes two settings (A and B), according to different clinical and care loads. On the basis of different endowments of nursing staff and monitoring equipment, the 2A setting is distinguished for patients with a greater clinical care load (acute neurological disease plus impairment of another vital organ) and the 2B setting is intended for other patients. The 2A setting provides for complete multi-professional/multi-specialist care and dedicated healthcare personnel (doctors, nurses, and health care assistant). In the 2A setting, a higher endowment of nursing and health care assistant is foreseen than in level 2B (with a nurse-to-patient ratio of 1:6–1:8). This also allows a slight reduction in costs compared with a sub-intensive ward and classical stroke unit (1:4).

The 2A setting of Stroke Unit/Neurocritical Care of the Santo Stefano Hospital in Prato (USL Toscana Centro) provides care and treatment specifically to patients suffering from cerebrovascular diseases and other AND.

### Stroke Unit/Neurocritical Care (2A setting) of the Santo Stefano Hospital (Prato, USL Toscana Centro): a pilot model

In this paper, we report the pilot experience of Stroke Unit/Neurocritical Care (2A) of the Santo Stefano Hospital (Prato, USL Toscana Centro).

The neurology department in the Santo Stefano Hospital consists of 30 beds. The 2A setting of Stroke Unit/Neurocritical Care includes 15 beds; 8 ± 2 beds are monitored by portable multi-parameter monitoring devices. In the Stroke Unit/Neurocritical Care (2A), patients undergo acute phase treatment and subsequently diagnostic and etiopathogenetic exams with continuous monitoring of vital functions, as well as secondary prevention therapy. Moreover, patients undergo early rehabilitation and prevention of complications beyond anti-immobilization strategies.

We retrospectively assessed the diagnoses in the hospital discharge forms (HDF) of Stroke Unit/Neurocritical Care (2A) of 249 patients consecutively analyzed between 1st January 2022 and 30th June 2022.

From a methodological point of view, we considered diagnoses of the main AND, considering in particular: ischemic stroke, hemorrhagic stroke, transient ischemic attack (TIA), cerebral vein thrombosis, epilepsy (first epileptic seizures), epileptic status, TBI (post-traumatic subdural hematoma, epidural hematoma, and subarachnoid hemorrhage), acute inflammatory polyradiculoneuritis, acute encephalitis, acute myelitis, acute myasthenia gravis, and others.

To be included in this report, patients had to be diagnosed as affected by the pathologies listed above according to the standard neurological definition, and confirmed on neuroimaging (CT scan or cerebral MRI) or neurophysiological standard criteria. Regarding ischemic strokes, we considered intravenous fibrinolytic and intra-arterial treatment; our patients are treated with endovascular treatment at the AOU Careggi Hospital, as the hub-center of USL Toscana Centro. Regarding hemorrhagic stroke, we considered typical and atypical intraparenchymal cerebral hemorrhage (according to ICH criteria, and on the basis of CT/MRI injury site) and spontaneous non-traumatic subarachnoid hemorrhage.

The assessment was retrospective and consecutive.

To illustrate the baseline total sampled descriptive analyses were used. Univariate statistical analyses (independent samples *t*-test for continuous variables) were used to compare the groups of TBI patients.

We screened 378 patients hospitalized in the 6-month period between 1st January 2022 and 30th June 2022 and consecutively selected among diagnoses of HDF of Stroke Unit/Neurocritical Care (2A).

**Figure 1:**
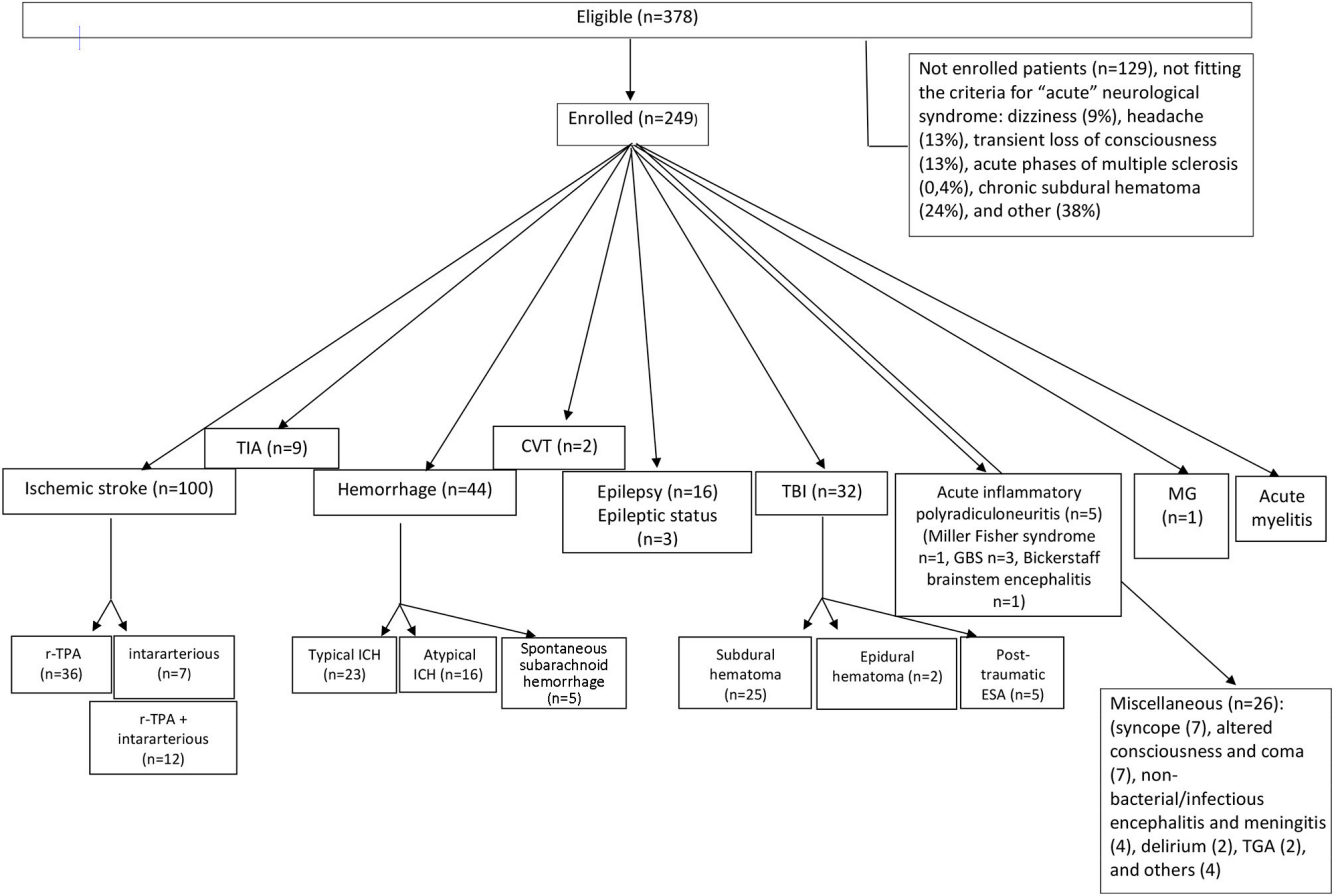
Flow diagram showing patients' distribution diagnosis in the 2A setting of Stroke Unit/Neurocritical Care in the Santo Stefano Hospital (Prato, USL Toscana Centro). TIA, transient ischemic attack; ICH, intraparenchymal cerebral hemorrhage; CVT, cerebral venous thrombosis; TBI, traumatic brain injury; GBS, Guillain-Barré syndrome; MG, myasthenia gravis; TGA, transient global amnesia.

We excluded 129 patients (34%) because they did not fit the criteria for “acute” neurological syndromes: dizziness (*n* = 11, 9%), headache (*n* = 16, 13%), transient loss of consciousness (*n* = 16, 13%), multiple sclerosis (*n* = 5, 0.4%), chronic subdural hematoma (*n* = 31, 24%), and others (*n* = 50, 38%). Figure 1 shows the main exclusion criteria.

According to the international standard diagnostic criteria, out of the 249 patients affected by AND, 155 had cerebrovascular diseases (62.2%). In particular, 100 (64.5%) were diagnosed as ischemic stroke, nine (5.8%) as TIA, 44 (28.4%) as hemorrhagic stroke, and two (1.2%) as affected by central venous thrombosis (Figure 1).

Out of the 100 patients with diagnosis of acute ischemic stroke, 36 (36%) were treated with r-TPA, seven (7%) patients with endovascular treatment, and 12 (12%) underwent r-TPA combined with intararterious thrombectomy; 45 (45%) patients were outside the therapeutic window for thrombolytic treatment and treated with antiaggregation therapy (Figure 1). One stroke patient (1%) was intrahospital stroke. The setting discharge of the cerebrovascular patients were: rehabilitation (28%), intermediate care hospitals (31%), long-term care (6%), and home (35%).

Among the main classical ethiopathogenic causes of stoke, 30% were cardioembolic, 24% atherosclerotic, 38% lacunar, and 8% were embolic stroke of undetermined source (ESUS); one patient had diagnosis of Mitochondrial myopathy, Encephalopathy, Lactic Acidosis and Stroke-like episodes Syndrom (MELAS), and two patients of venous thrombosis of the central nervous system.

Out of the 44 patients with hemorrhagic stroke, 23 (52.3%) had a hemorrhage in a typical area of hypertensive microangiopathy, while 16 (36.4%) hemorrhages were atypical. Five (11.3%) patients had primarily spontaneous subarachnoid hemorrhages (Figure 1).

Regarding some outcome data, analyzing the residual disability of the cerebrovascular events in out cohort, the 3-month modified Rankin Scale (mRS) in 36 stroke patients treated with r-TPA was 0–2 (low disability) in 60% of cases, 3–4 (moderate-severe disability) in 35%, and 5–6 (severe disability/death) in 5% of patients. In 64 non-treated patients, the mRS was 0–2 in 21%, 3–4 in 53%, and 5–6 in 26% of patients.

Out of the 44 ICH patients, 31% reported severe disability or death, 47% moderate-severe degree of disability (3–4), and 22% 0–2 mRS.

Thirty-two patients (32/249, 12.8%) were hospitalized following a TBI; 25 (78%) for post-traumatic subdural hematoma, two (6.25%) for acute post-traumatic epidural hematoma, and five (15.6%) for post-traumatic subarachnoid hemorrhage (Figure 1) (underlying causes are available upon request).

Taking into account the 30 TBI patients hospitalized in different departments (geriatric, medicines, and intensive therapy) for comparison, the mRS range was from 1 to 5 for all TBI patients, with differences between the patients in Stroke Unit/Neurocritical Care (2A) and those in other departments (mRS 1–3 vs. mRS 3–4, respectively).

Sixteen patients (6.4% of all AND) were hospitalized for a new diagnosis of epilepsy and three (1.2%) for epileptic status (Figure 1) (underlying causes are available on request).

Out of the other AND, acute inflammatory polyradiculoneuritis was found in five patients; of these, one had a Miller Fisher syndrome, three had Guillain–Barré syndrome, and one had Bickerstaff brainstem encephalitis (Figure 1). One patient had acute crisis of myasthenia gravis, and one had acute myelitis (Figure 1). The remain patients (*n* = 26) were hospitalized for: syncope (7), altered consciousness and coma (7), non-bacterial/infectious encephalitis and meningitis (4), delirium (2), transient global amnesia (TGA) (2), and others (4) (Figure 1).

The detailed distributions of patients are shown in Figure 1.

Globally, the setting discharge of all AND patients were: rehabilitation (26%), intermediate care hospitals (44%), long-term care (5%), and home (25%).

Regarding other summary outcome data, the one-month mortality rate in Stroke Unit/Neurocritical Care (2A) patients was 1.8%.

Moreover, the percentages of re-entry to hospital of patients admitted to our setting was around 17% (generally due to infectious causes and not recurrences of the primary cause of hospitalization).

## Discussion and recommendations

We reported a pilot model of the Stroke Unit/Neurocritical Care (2A setting) of the Santo Stefano Hospital (Prato, USL Toscana Centro).

In particular, this report analyzed 378 patients hospitalized in a 6-month period in our hospital, half of whom (249) because of neurological pathologies were defined as acute (AND). Hospitalization volumes in our hospital are considerable, especially because it is a small hospital. Moreover, the series of patients is very varied and interesting. Because our department was originally a stroke unit, more than 60% of patients had cerebrovascular diseases.

We provided a brief description of the cases treated in 6-month to draw attention to the possibility of the existence of a ward dedicated exclusively and specifically to all AND (and all neurological pathologies in acute phases), as our Stroke Unit/Neurocritical Care. As an intensive care unit, patients are monitored continuously and immediately treated for possible complications. But unlike an intensive care unit, patients are thoroughly studied with particular regard to the underlying etiopathogenesis and secondary prevention therapies.

The data and descriptive analysis shown are substantially in line with literature data about acute neurological pathologies (World Health Organization, 2018a,b; Shrestha and Lamsal, 2020; Prust et al., 2022).

As expected, regarding the residual disability of stroke patients, the mRS was good when treated with r-TPA and hospitalized in our setting. Our data are consistent with the literature data of patients treated in stroke units compared with non-specialized wards, with mortality reduced (absolute risk reduction) by 3%, dependency reduced by 5%, and institutionalization reduced by 2 % (Langhorne and Ramachandra, 2020). Data from the PROSIT study showed a 19% decrease in mortality and residual disability in patients admitted to stroke units, with 1 patient with mRS 0–2 for every 100 treated (Candelise et al., 2007). In the future, systematic analysis with systematized outcome data of our setting will be provided.

Interestingly, we reported a rough exploratory analysis of the mRS comparison between TBI admission in Stroke Unit/Neurocritical Care (2A) and in other departments, with a clear advantage in favor of our setting. Moreover, global mortality data for this sample are low.

There are a few considerations related to this pilot model that can be raised.

First, according to WHO, “in settings where clinical demand far exceeds available critical care resources, the principle of maximizing clinical benefit for the patient population must be weighed respect to allocating care” (World Health Organization, 2018a). Some data exposed above demonstrate that there is scientific evidence of the best quality of care in stroke units in the treatment of cerebrovascular disease, with substantial improvements in mortality and disability (Prust et al., 2022). Moreover, there are also some studies, although few, that showed advantages in terms of assistance also in neurocritical care units, especially for trauma and TBI (Varelas et al., 2004; Suarez, 2006; Kramer and Zygun, 2014; Shrestha and Lamsal, 2020). The worldwide burden of AND and the need of specialist neurocritical care skills and specialized neurocritical care for managing neurological emergencies has increased (Prust et al., 2022). The benefit of the diagnostic-therapeutic strategies we apply, such as monitoring of vital functions, prevention and treatment of complications, and rehabilitation strategies, have already been demonstrated in stroke units and in neurocritical care units (Varelas et al., 2004; Suarez, 2006; Kramer and Zygun, 2014; Shrestha and Lamsal, 2020; Prust et al., 2022). In our setting (2A), beyond acute treatment protocols, some standardized procedures were organized, in particular: routine advanced modalities of multiparametric non-invasive monitoring of vital signs, early access to neuroimaging and to neurophysiological investigations, early treatment of complications and seizures, prompt treatment of fevers and hypoxia, prevention of complications such as the adoption of early mobilization strategies and prevention of thromboembolism, routine management of dysphagia, aspiration pneumonia, and prompt mechanical ventilation.

Second, the presence of medical doctors and nursing staff with specific neurocritical care training guarantees the best possible assistance for acute neurological patients. In the stroke unit, neurocritical care unit staff have a specialist interest in stroke and AND and provide a coordinated multidisciplinary rehabilitation package with team training and education, and standard issue management protocols (Langhorne, 2021). In this context, in our opinion, training programs should be implemented to improve skills in care in neurocritical settings so as to guarantee a greater number of qualified professionals within critical health systems in order to increase the quality of patient care, with savings of long-term resources.

Thirdly, it is important to underline the importance of neurophysiological investigation in AND. Rapid access to neurophysiological investigation, as EEG, continuous EEG, and electromyography, is crucial to the diagnosis and treatment of, respectively, epileptic seizure, convulsive/non-convulsive epileptic status, and acute inflammatory polyradiculoneuritis.

Fourthly, the inpatient registry (such as HDF) we used and analyzed can support physicians for future management of acute neurological diseases in limited settings. Hospital-based registries of inpatient neurologic diseases are necessary and have been shown to be feasible in resource-limited settings (Langhorne and Dennis, 1998; Langhorne, 2021; Nutakki et al., 2021). A concerted effort from physicians working in neurocritical care to perform high-quality research to better understand local disease patterns and treatment responses is also clearly needed (Ringelstein et al., 2013).

Our study has several limitations: (a) the small sample size (especially for the subgroups) precluded us from performing multivariate analyses; (b) the lack of a control group (except in the TBI subgroup) restricted comparison analyses; (c) there was a paucity of outcome data, mostly descriptive only. This is an important limitation, because we showed few available outcome data regarding mRS in some acute neurological patients; this is because systematic outcome information was not available for all the pathologies considered. However, outcome data are a big issue in general because they are not implemented in clinical practice, except in selected studies (Ebrahim, 1990; Suarez, 2006). Outcome data on large cohorts of patients are very limited and generally not systematically used. Beyond the local level, strengthening global networks for research and data sharing is likely to broaden the reach of individual investigators and accelerate the innovation of care (Schwalbe et al., 2020). Hospital-based registries are needed.

In conclusion, our pilot model seems to suggest some improvements in patient function and outcomes, measurable for example with the mRS. Our data must be confirmed in a longitudinal study exploring the outcome of AND patients. This report about Stroke Unit/Neurocritical Care (2A setting) of the Santo Stefano Hospital, although with a small number of outcomes, shows high quality care and treatment in patients affected by strokes and other important AND. Future work focusing on long-term prognosis is needed.

## Data availability statement

The original contributions presented in the study are included in the article/supplementary material, further inquiries can be directed to the corresponding author.

## Author contributions

RV wrote the paper. PP implemented the setting model. All authors contributed to work in the clinical setting. All authors contributed to the article and approved the submitted version.
